# Land-Jump Performance in Patients with Juvenile Idiopathic Arthritis (JIA): A Comparison to Matched Controls

**DOI:** 10.1155/2009/478526

**Published:** 2010-01-27

**Authors:** Kevin R. Ford, Gregory D. Myer, Paula G. Melson, Shannon C. Darnell, Hermine I. Brunner, Timothy E. Hewett

**Affiliations:** ^1^Sports Medicine Biodynamics Center and Human Performance Laboratory, Cincinnati Children's Hospital Research Foundation, Cincinnati, OH 45229, USA; ^2^Department of Pediatrics, College of Medicine, University of Cincinnati, Cincinnati, OH 45221, USA; ^3^Rocky Mountain University of Health Professions, Provo, UT 84606, USA; ^4^Division of Rheumatology, Cincinnati Children's Hospital, Cincinnati, OH 45229, USA; ^5^Division of Occupational and Physical Therapy, Cincinnati Children's Hospital, Cincinnati, OH 45229, USA; ^6^Department of Orthopaedic Surgery, College of Medicine, University of Cincinnati, Cincinnati, OH 45219, USA

## Abstract

*Objective*. The purpose of this study was to determine if high functioning children with Juvenile Idiopathic Arthritis (JIA) with minimal disease activity have different biomechanics during high loading tasks compared to controls. Patients were included if they had minimal inflammation documented in one or both knees. Methods. The subject groups consisted of eleven patients with JIA and eleven sex, age, height, and weight matched controls. Sagittal plane kinematic and kinetics were calculated during a drop vertical jump maneuver. The Child Health Assessment Questionnaire (CHAQ) was collected on each patient with JIA. Results. The subjects with JIA had increased knee (*P* = .011) and hip flexion (*P* < .001) compared to control subjects. Subjects with JIA also demonstrated decreased knee extensor moments during take-off (*P* = .028) and ankle plantar flexor moments during landing (*P* = .024) and take-off (*P* = .004). In the JIA group, increased hip extensor moments were predictive of increased disability (*R* ^2^ = .477, *S* *E* *E* = .131). *Conclusions*. Patients with JIA may demonstrate underlying biomechanical deviations compared to controls. In addition, biomechanical assessment of hip extensor mechanics during dynamic tasks may provide an objective assessment tool to determine overall function in patients with JIA.

## 1. Introduction

Juvenile Idiopathic Arthritis (JIA) is a childhood disease characterized by chronic, recurrent inflammation of the joints [1]. Although JIA is rarely a life-threatening condition, it can affect growth and is associated with disability, increased need for joint arthroplasty, and decreased quality of life. Articular effusion, synovial hypertrophy, stiffness, and pain are hallmarks of the disease [2]. The mechanical effects of these abnormalities, compounded by the potentially erosive effects of the inflammatory process, can lead to transient or long-term musculoskeletal defects [3]. Delayed neuromuscular development, muscular weakness, ligamentous laxity and generalized or localized growth disturbances can all be factors that contribute to musculoskeletal changes [4]. Historically, patients with JIA have been managed with medication and conservative exercise. The condition usually requires long-term drug therapy treatment and may result in at least some restrictions of physical activity, which depend on the type and severity of the disease. The immediate effects of improved physical function and decreased pain in patients with JIA are decreased self-limitation of functional activities and potentially increased involvement in recreational or competitive sports. 

JIA is characterized by periods of quiescence and exacerbation. In times of quiescence, when patients may have normal clinical measures of range of motion and strength, the underlying disease is still present, which results in difficult decisions about participation in high-impact activities. Frequently, the activities that patients request to participate in are basketball, volleyball, and soccer. Although pain may be absent, the potential presence of underlying biomechanical and neuromuscular deficits and ultimately the long-term implications of high-impact loading are unknown. Abnormal joint mechanics in young athletes may be associated with an increased risk of sports injuries. Abnormal joint use may consist of decreased knee joint moments or increased moments at the ankle and hip. In order to investigate the risks and benefits of high-level sports participation in children with JIA, knowledge should first be obtained about the characteristic lower extremity joint mechanics in times of disease control. The purpose of the current study was to determine if high functioning children with JIA with minimal disease activity have different biomechanics during high loading tasks and whether these biomechanical measures relate to subjective measures of function. The first hypothesis was that patients with JIA would demonstrate decreased peak knee joint moments compared to matched controls. The second study hypothesis was that increased ankle and hip joint moments would be related to subjective functional disability measures in the patient group.

## 2. Materials and Methods

### 2.1. Subjects

The study cohort consisted of eleven patients with JIA who had documented minimal inflammation in one or both knees on pediatric rheumatologist exam at the time of enrollment (4 male, 7 female; age: 13.0 ± 2.6 years; height: 154.7 ± 14.1 cm; mass: 45.0 ± 12.2 kg). Excluded were patients with JIA who had range of motion limitations as measured goniometrically in hips, spine, knees, ankles, or shoulders, as well as those with active inflammation of the hips, ankles, and spine. Eight patients when enrolled in the study had oligoarticular joint involvement and three had polyarticular joint involvement. 

As expected, the patients had a low level of physical functional deficit with a mean Child Health Assessment Questionnaire (CHAQ) score of 0.148 (range 0–0.5). The CHAQ is a commonly used outcome measure in the field of pediatric rheumatology and has been previously determined to be both valid (Kendall's tau *b* = 0.77, *P* < .0001) and reliable (internal reliability: Cronbach's *α* = 0.94, test-retest: *P* > .9) [5]. The disability index utilizes thirty items to assess function in eight areas—dressing and grooming, arising, eating, walking, hygiene, reach, grip, and activities. Possible scores range from 0.0 (optional functional status) to 3.0 (severe disability) [5].

Eleven healthy control subjects without a history of lower extremity joint disease were tested. Controls were matched to JIA subjects according to sex, age, height, and weight (Controls: age: 12.9 ± 1.9 years; height: 157.0 ± 13.1 cm; mass: 40.7 ± 15.6 kg). The 11 JIA patients had a total of 16 involved knees with minimal arthritis. Each lower extremity with an affected knee was considered separately for the purposes of the analysis and was compared with 16 matched lower-extremities (based on dominant and nondominant limb) of the control group. All subjects parents or guardians read and signed the informed written consent, approved by the Cincinnati Children's Hospital Institutional Review Board and in compliance with the Helsinki Declaration, prior to participation. 

### 2.2. Procedures

Each subject was instrumented with 37 retroreflective markers placed on the sacrum, left posterior superior iliac spine (PSIS), sternum and bilaterally on the shoulder, elbow, wrist, anterior superior iliac spine (ASIS), greater trochanter, mid thigh, medial and lateral knee, tibial tubercle, mid shank, distal shank, medial and lateral ankle, heel, dorsal surface of the midfoot, lateral foot (5th metarsal) and toe (between 2nd and 3rd metatarsals). A static trial was first collected in which the subject was instructed to stand still and was aligned with the laboratory coordinate system. This measurement was used as each subject's neutral (zero) alignment; subsequent kinematic measures were in relation to this position. 

The subjects were shown the drop vertical jump and allowed to practice the maneuver. The drop vertical jump involved the athlete dropping off a 31 cm box and immediately performing a maximum vertical jump. Three trials were collected for analysis. 

### 2.3. Instrumentation

Movement was recorded using a motion analysis system consisting of eight digital cameras (Eagle cameras, Motion Analysis Corporation, Santa Rosa, CA) sampling at 240 Hz. Two force platforms (AMTI, Watertown, MA) were sampled at 1200 Hz and time synchronized with the motion analysis system. The force platforms were positioned so each foot would contact a separate platform during the DVJ trials. 

### 2.4. Data Analysis

**Figure 1:**
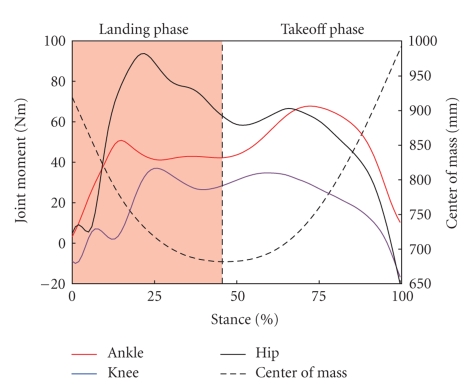
Lower extremity joint moments from one subject illustrating identification of landing and takeoff phases during the drop vertical jump maneuver. Phases were divided based on the lowest point of the body's center of mass. Maximum extensor moments were determined for the ankle, knee and hip during each phase.

Data were collected with EVaRT (Version 4.2, Motion Analysis Corporation) and imported into KinTrak (Version 6.2, Motion Analysis Corporation) for data reduction and analysis. The three-dimensional Cartesian marker trajectories were filtered through a second-order low-pass Butterworth digital filter at a cutoff frequency of 15 Hz. Ankle and knee joint centers were defined as the midpoint between the medial and lateral ankle and knee markers, respectively. The hip center was identified based on the greater trochanter and ASIS markers [6]. A joint coordinate system was defined within KinTrak [7] with the proximal and distal segments of each joint (6 degrees of freedom). Ankle, knee, and hip flexion-extension range of motion from initial contact to the maximum angle during the initial stance phase (landing off the box) was calculated. Joint moments were calculated from the motion and force data using a standard inverse dynamics approach within KinTrak. The force data were also filtered at the same cutoff frequency of 15 Hz in order to minimize possible impact peak errors during the calculation of joint moments [8]. Net internal moments are described in this paper and represent the body's response to the external load on the joint. Maximum extensor moments were calculated during both the landing and take-off phases of the drop vertical jump maneuver and normalized to percent of body weight (Newton) multiplied by height (meter). The landing phase was defined as when the body's center of mass was decelerating and the take-off phase was when the body's center of mass was accelerating (Figure 1). Total contact time and the distribution of phases were not different between groups. 

### 2.5. Statistical Analyses

Means and standard deviations for each variable based on the three trials performed were calculated for each subject. A one-way MANOVA was utilized to determine the effect of JIA on the dependent variables (*P* < .05). A *post-hoc* univariate analysis was then performed to determine whether the knee joint moments were decreased and ankle and hip increased in the patient population compared to controls. Joint range of motion was also examined to determine if the patients with JIA had increased range of motion compared to the controls. Stepwise multiple linear regression was utilized to determine which biomechanical variables significantly predict the CHAQ score (criteria *F* < 0.100). Statistical analyses were conducted in SPSS (SPSS, Version 12.0, Chicago, IL). 

## 3. Results

**Figure 2:**
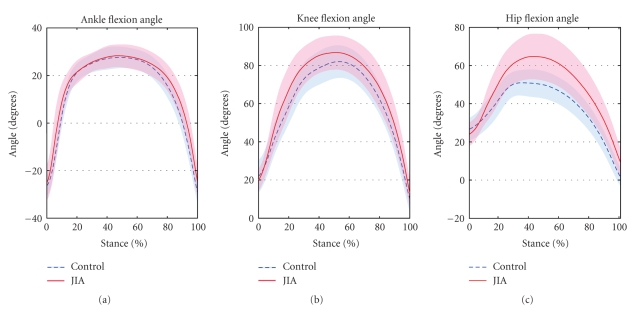
Ensemble averaged (mean ± 1 standard deviation) lower extremity joint angles during the drop vertical jump.

A significant difference between JIA and controls was found when all the dependent variables within the MANOVA were examined. (*F*_(9,22)_ = 2.7, *P* = .027). There were no differences in the dependent variables (hip, knee, and ankle ROM and moments) between the unilaterally and bilaterally involved patients (*F*_(9,6)_ = 1.2, *P* = .44). Figure 2 shows the ankle, knee, and hip sagittal plane angles during the DVJ for each group. Involved JIA limbs had significantly greater range of motion at the knee (*P* = .011) and hip (*P* < .001) compared to the matched controls (Table 1). There were no differences between groups in ankle range of motion (*P* = .329).

**Figure 3:**
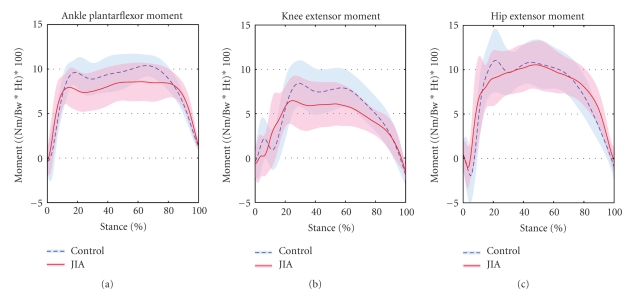
Ensemble averaged (mean ± 1 standard deviation) lower extremity joint moments during the drop vertical jump.

Lower extremity joint moments are presented in Figure 3. The knee joint moment during the take-off phase was significantly decreased in the JIA group compared to the controls (*P* = .028). Knee joint moments were not different between groups during the initial landing phase (*P* = .122). The ankle plantar flexor moments were significantly decreased in the JIA groups during both the landing and take-off phases (*P* = .024, *P* = .004) of the drop vertical jump when compared to controls (Table 1). Groups did not differ in hip extensor moments during either phases of the drop vertical jump (Table 1). 

In order to examine the potential biomechanical predictors of CHAQ (physical function or disability), a stepwise multiple linear regression was performed. The only statistically significant predictor of the CHAQ was the hip extensor moment during landing. Hip extensor moments alone correlated to CHAQ with an *R* value of 0.69 and predicted 48% of the variability of the CHAQ score (*R* ^2^ = 0.48, SEE = 0.131). In patients with JIA, increased hip extensor moments were predictive of a poor CHAQ score.

## 4. Discussion

The purpose of the current study was to determine if high functioning children with JIA with minimal disease activity have different biomechanics during high loading tasks and whether these biomechanical measures relate to subjective measures of function. JIA is a chronic autoimmune disease characterized by periods of quiescence and exacerbation. In times of quiescence, when patients appear to have normal or near normal clinical measures of range of motion and strength, the underlying disease is still present, which results in difficult decisions about participation in high-impact activities. Therefore, the investigation of a landing and jumping activity in these well-controlled, high functioning patients is warranted to compare with matched control athletes. 

Patients with JIA did exhibit decreased knee joint moments during the take-off phase of the DVJ, partially supporting the first study hypothesis. These findings may indicate that the patients transfer the joint moments to other lower extremity joints. However, increased joint moments at the ankle and hip were not found in the JIA group compared to controls in the current study. It is possible that the decreased knee extensor moment may be indicative of quadriceps weakness. Further evaluation of lower extremity strength measures in patients with JIA may help determine if knee extensor strengthening exercises are warranted in this population. In addition, overall performance (i.e., vertical jump height) may also be an important variable to investigate in this population. 

Patients with JIA demonstrated increased range of motion on the involved side at the knee and hip compared to the control group. Increased sagittal plane range of motion may be an adaptive strategy used by patients with JIA to reduce ground reaction forces and joint loads. In the current study, these patients appeared to have adapted an increased sagittal plane motion strategy to control joint loading. Similar landing strategies have been identified and termed either a soft, flexion style landing or a stiff, bounce landing [9, 10]. The “softer” type of landing appeared to be adopted by the patients with JIA. While this landing strategy does not necessarily indicate a “safer” or less injurious style of landing, it may be a neuromuscular adaptation, due in part to the disease history. Whether this may translate into a higher or lower risk of injury is unknown.

With regard to kinetic measures, significant differences during the landing phase were found between patients and controls in ankle plantar flexor moments. The patients with JIA exhibited decreased ankle torques compared to controls. Decreased ankle moments were unexpected in the patients with JIA, since prior studies indicated that patients with symptomatic or postoperative knees utilized increased joint loading patterns at adjacent joints. Specifically, patients with reconstructed knees increased ankle joint loads by nearly 40% [11]. However, Devita and Skelly [10] found that soft style landing was associated with decreased ankle plantar flexor moments, without changes in knee and hip extensors, when compared to stiff landings. Therefore, the mechanisms behind the decreased ankle moments in the current study group appeared to relate to different landing mechanics. 

In the JIA group, as the CHAQ score increased (indicative of increased functional deficits) the hip extensor moments also increased during landing. These findings indicate that the patients with higher disability utilize increased hip strategies during dynamic tasks. This partially supports the second study hypothesis, since patients with decreased function appeared to adapt with greater hip extensor torque during the landing. Objective assessment of hip dynamics may be a useful tool to aid clinicians in the determination of functional status and may be helpful for making activity restrictions related to sports participation. A direct link between injury risk and altered landing mechanics in this patient population is difficult to ascertain. Follow-up studies with a larger range of CHAQ scores may be necessary to further develop the relationship between the hip and poor function. However, this study does appear to be an important step towards developing functional outcome measures in children with JIA. Future investigations should examine the relationship between injury risk and landing mechanics in patients with JIA.

## Figures and Tables

**Table 1:** Mean (SD) kinematic and kinetic variables for the involved sides of the JIA and Control groups. Multivariate analysis was significantly different between groups (*F*_(9,22)_ = 2.7, *P* = .027). Univariate *post-hoc* analysis is listed for each variable (*P < .05).

	JIA	Control	Univariate
*Kinematics—Angular ROM*			
Ankle (°)	53.8	55.2	*F*_(1,30)_ = 0.2
	(10.3)	(5.8)	*P* = .329
Knee (°)	*67.5	*60.5	*F*_(1,30)_ = 6.0
	(8.8)	(7.5)	*P* = .011
Hip (°)	*41.5	*25.4	*F*_(1,30)_ = 22.8
	(12.5)	(5.0)	*P* < .001

*Kinetics—Landing*			
Ankle Moment ((Nm/(bw*ht))*100)	*10.2	*11.3	*F*_(1,30)_ = 4.2
	(1.6)	(1.3)	*P* = .024
Knee Moment ((Nm/(bw*ht))*100)	8.5	9.7	*F*_(1,30)_ = 1.4
	(2.5)	(3.3)	*P* = .122
Hip Moment ((Nm/(bw*ht))*100)	13.4	13.1	*F*_(1,30)_ = 0.1
	(2.8)	(2.6)	*P* = .380

*Kinetics—Take-off*			
Ankle Moment ((Nm/(bw*ht))*100)	*9.8	*11.2	*F*_(1,30)_ = 8.3
	(1.6)	(1.2)	*P* = .004
Knee Moment ((Nm/(bw*ht))*100)	*7.0	*8.4	*F*_(1,30)_ = 4.0
	(1.8)	(2.2)	*P* = .028
Hip Moment ((Nm/(bw*ht))*100)	11.3	11.7	*F*_(1,30)_ = 0.2
	(2.3)	(2.3)	*P* = .332

## References

[1] Petty RE, Southwood TR, Manners P (2004). International League of Associations for Rheumatology classification of juvenile idiopathic arthritis: second revision, Edmonton, 2001. *Journal of Rheumatology*.

[2] Emery HM, Bowyer SL, Sisung CE (1995). Rehabilitation of the child with a rheumatic disease. *Pediatric Clinics of North America*.

[3] Hafner R, Truckenbrodt H, Spamer M (1998). Rehabilitation in children with juvenile chronic arthritis. *Bailliere’s Clinical Rheumatology*.

[4] Klepper SE (2003). Exercise and fitness in children with arthritis: evidence of benefits for exercise and physical activity. *Arthritis and Rheumatism*.

[5] Singh G, Athreya BH, Fries JF, Goldsmith DP (1994). Measurement of health status in children with juvenile rheumatoid arthritis. *Arthritis and Rheumatism*.

[6] Bell AL, Pedersen DR, Brand RA (1990). A comparison of the accuracy of several hip center location prediction methods. *Journal of Biomechanics*.

[7] Stefanyshyn DJ, Stergiou P, Lun VMY, Meeuwisse WH, Worobets JT (2006). Knee angular impulse as a predictor of patellofemoral pain in runners. *American Journal of Sports Medicine*.

[8] van den Bogert AJ, de Koning JJ On optimal filtering for inverse dynamics analysis.

[9] Bobbert MF, Huijing PA, van Ingen Schenau GJ (1987). Drop jumping. I. The influence of jumping technique on the biomechanics of jumping. *Medicine and Science in Sports and Exercise*.

[10] DeVita P, Skelly WA (1992). Effect of landing stiffness on joint kinetics and energetics in the lower extremity. *Medicine and Science in Sports and Exercise*.

[11] Decker MJ, Torry MR, Noonan TJ, Riviere A, Sterett WI (2002). Landing adaptations after ACL reconstruction. *Medicine and Science in Sports and Exercise*.

